# Do outcomes for patients with hospital-acquired Acute Kidney Injury (H-AKI) vary across specialties in England?

**DOI:** 10.1186/s12882-023-03197-z

**Published:** 2023-06-29

**Authors:** Winnie Magadi, Javeria Peracha, William S McKane, Manuela Savino, Fiona Braddon, Retha Steenkamp, Dorothea Nitsch

**Affiliations:** 1 UK Renal Registry, UK Kidney Association, Bristol, UK; 2 Department of Nephrology, The Royal Wolverhampton NHS Trust, Wolverhampton, UK; 3 Sheffield Kidney Institute, Sheffield Teaching Hospitals NHSFT, Sheffield, UK; 4 Acute Internal Medicine, Great Western Hospitals NHS Foundation Trust, Swindon, UK; 5 Faculty of Epidemiology and Population Health, London School of Hygiene and Tropical Medicine, London, UK; 6 Department of Nephrology, Royal Free London NHS Foundation Trust, London, UK; 7 UK Renal Registry, Brandon House 20a1, Southmead Road, Bristol, BS34 7RR UK

**Keywords:** Acute Kidney Injury, Mortality risk, Treatment specialty

## Abstract

**Background:**

Acute Kidney Injury (AKI) is a common and serious clinical syndrome. There is increasing recognition of heterogeneity in observed AKI across different clinical settings. In this analysis we have utilised a large national dataset to outline, for the first time, differences in burden of hospital acquired AKI (H-AKI) and mortality risk across different treatment specialities in the English National Health Service (NHS).

**Methods:**

A retrospective observational study was conducted using a large national dataset of patients who triggered a biochemical AKI alert in England during 2019. This dataset was enriched through linkage with NHS hospitals administrative and mortality data. Episodes of H-AKI were identified and attributed to the speciality of the supervising consultant during the hospitalisation episode in which the H-AKI alert was generated. Associations between speciality and death in hospital or within 30 days of discharge (30-day mortality) was modelled using logistic regression, adjusting for patient age, sex, ethnicity, socioeconomic status, AKI severity, season and method of admission.

**Results:**

In total, 93,196 episodes of H-AKI were studied. The largest number of patients with H-AKI were observed under general medicine (21.9%), care of the elderly (18.9%) and general surgery (11.2%). Despite adjusting for differences in patient case-mix, 30-day mortality risk was consistently lower for patients in surgical specialities compared to general medicine, including general surgery (OR 0.65, 95% CI 0.61 to 0.7) and trauma and orthopaedics (OR 0.52, 95% CI 0.48 to 0.56). Mortality risk was highest in critical care (OR 1.78, 95% CI 1.56 to 2.03) and oncology (OR 1.74, CI 1.54 to 1.96).

**Conclusions:**

Significant differences were identified in the burden of H-AKI and associated mortality risk for patients across different specialities in the English NHS. This work can help inform future service delivery and quality improvement activity for patients with AKI across the NHS.

**Supplementary Information:**

The online version contains supplementary material available at 10.1186/s12882-023-03197-z.

## Introduction

Acute kidney injury (AKI) is characterised by a sudden reduction in kidney function. It is extremely common in hospital settings, affecting up to a fifth of admitted patients [1–3]. Common precipitants for AKI include sepsis, dehydration, cardiac failure, nephrotoxic medication use, major surgery or trauma [4]. Vulnerable patients are cared for across a diverse range of hospital specialities [5, 6]. Patients who develop AKI are at high risk of serious complications including death, kidney failure requiring dialysis, prolonged hospitalisation, emergency readmission, chronic kidney disease (CKD) and major adverse cardiovascular events [7–9]. Previously, reports such as the ‘National Confidential Enquiry into Patient Outcomes and Death’ (NCEPOD) for patients with AKI in England (2009), highlighted concerns regarding poor care for patients with AKI across the National Health Service (NHS), contributing to instances of avoidable patient harm [10]. A more recent national audit examining the quality of care received by patients hospitalised with moderate and severe AKI in 2019 raised ongoing concerns about unwarranted variation and deficiencies in NHS organisations achieving key AKI process of care quality metrics [11]. As such, improving care for patients with AKI remains a national patient safety priority in England [12, 13]. Routine monitoring and comparison of AKI patient outcomes across different healthcare providers can serve as a valuable quality improvement tool to support this work. To this effect, in England we have now established a national dataset of laboratory identified patients with biochemical evidence of AKI, using the NHS AKI algorithm [14]. This dataset is known as the ‘Master Patient Index’ (MPI) and is routinely linked to NHS hospitals administrative data (Hospital Episodes and Statistics, HES) and mortality feeds from the Office for National Statistics (ONS) [15, 16].

There were over 500,000 cases of AKI in England identified using the MPI during 2018 [16]. There is likely to be considerable heterogeneity in these AKI cases, influenced by the underlying cause for AKI, patient factors, treatment and clinical setting [17]. Previously, we have reported development of a provider-level standardised mortality metric for all patients with hospital acquired AKI (H-AKI) across NHS Trusts in England [15]. Further exploration of trends in H-AKI rates and outcomes between patient subgroups may facilitate better phenotyping of those most at risk of developing this syndrome and associated poor outcomes. In this study, we utilised the national MPI-HES dataset to explore, for the first time, differences in the number of patients with H-AKI and 30-day mortality risk across different specialities in England.

## Methods

### Study design

This was a retrospective observational study comprising all adults (> 18 years) with hospital acquired AKI between 01/01/2019 to 31/12/2019, at acute healthcare organisations (termed ‘trusts’ in the NHS) in England. H-AKI was defined as an AKI episode first detected more than two days after the date of hospital admission [18]. All patients had follow-up until death or 30 days after discharge.

### Dataset

This study used the MPI dataset linked to HES, held at the UK Renal Registry. The MPI includes data from laboratories in England on patients who have triggered AKI alerts, using the ‘NHS AKI Algorithm’ [19]. Briefly, the algorithm detects a rise in serum creatinine (SCr) from laboratory held baseline values (either lowest SCr in preceding 7 days or if this is unavailable then the median SCr from the preceding 8-365 days). AKI is graduated into three levels of severity; 1, 2 and 3, corresponding to a SCr rise of 1.5-2.0, 2.0–3.0 and > 3.0 times baseline values respectively [20]. Linked HES data allowed extraction of additional information on patient demographics, co-morbidities and hospitalisations. Mortality data was obtained from kidney centres and ONS Civil Registration’s data [21–23].

Of note, any patients in the MPI known to be on renal replacement therapies (RRT) at time of AKI alert are removed by UKRR during data cleaning. Further, regarding the data completeness, the MPI dataset does not have full coverage for England. Of the 137 labs submitting data for patients with an AKI alert in 2019, 118 sent 12 months’ worth of data while completeness was incomplete/variable for the remaining 19 labs [24].

### Exposures of interest and outcomes

The primary exposure in the study was ‘*treatment specialty group*’. These groups were defined using *“treatment specialty codes”* available in HES, which describe the specialty of the supervising consultant (lead clinician) during each period of care (see Table 1). We allocated each H-AKI episode in our cohort to a speciality. This was the speciality of the supervising consultant during the hospitalisation episode in which the H-AKI alert was generated. For patients that were transferred between specialities, H-AKI episodes that occurred within 2 days of transfer were attributed to the prior speciality.

**Table 1:** Treatment specialty groups matched by HES treatment specialty codes

TREATMENT SPECIALTY GROUP	HES TREATMENT SPECIALTY (code)
General Medicine	*General Medicine (300)*
Care of the elderly	*Geriatric Medicine (430)*
	*Stroke Medicine (328)*
Respiratory Medicine	*Respiratory Medicine (Thoracic Medicine) (340)*
Diabetes and Endocrinology	*Endocrinology (302)*
	*Diabetic Medicine (307)*
Neurology	*Neurology (400)*
Gastroenterology	*Gastroenterology (301)*
	*Hepatology (306)*
Rheumatology	*Rheumatology (410)*
General Surgery	*General Surgery (100)*
	*Colorectal Surgery (104)*
	*Hepatobiliary & Pancreatic Surgery (105)*
	*Upper Gastrointestinal Surgery (106)*
	*Plastic Surgery (160)*
Nephrology	*Nephrology (361)*
Cardiology	*Cardiology (320)*
Clinical Haematology	*Clinical Haematology (303)*
Gynaecology	*Gynaecology (502)*
Cardiothoracic Surgery	*Cardiac Surgery (172)*
	*Cardiothoracic Surgery (170)*
	*Thoracic Surgery (173)*
Critical Care	*Critical Care Medicine (192)*
Head and Neck surgery	*Neurosurgery (150)*
	*Ear, Nose & Throat (ENT) (120)*
	*Maxillo Facial Surgery (144)*
Oncology	*Medical Oncology (370)*
	*Clinical Oncology (previously Radiotherapy) (800)*
	*Gynaecological Oncology (503)*
	*Bone & Marrow Transplantation (308)*
Trauma and Orthopaedics	*Trauma & Orthopaedics (110)*
	*Spinal Surgery Service (108)*
Urology	*Urology (101)*
Vascular Surgery	*Vascular Surgery (107)*

HES – Hospital Episode Statistics

The outcome of interest was mortality in hospital or within 30 days of discharge, referred to as 30-day mortality.

### Covariates

Risk factors considered in the analysis included age, sex, ethnicity, deprivation, season of admission, elective or emergency admission, comorbidity score (AKI specific re-weighting of Charlson Comorbidity Index as detailed in Supplementary Table S1) and peak AKI stage (1, 2 or 3). Deprivation was based on the Index of Multiple Deprivation (IMD) which is the official measure of deprivation in England, calculated at neighbourhood level and comprising 7 domains; income, employment, health deprivation/disability, education/ skills, crime, barriers to housing/services and living environment. IMD scores have been categorised into five quintiles for our analysis (1 being the least deprived and 5 being the most deprived) [25].

A subgroup analysis was performed for patients in our cohort that had H-AKI in association with an ‘infection’ as their ‘primary diagnosis’. *(Supplementary Table S2).*

### Statistical modelling

All analyses were conducted using SAS 9.4 (SAS Institute, Cary, NC, USA) and Stata/MP12 (StataCorp, College Station, TX, USA). We ran logistic regression analyses to describe associations of treatment speciality with 30-day mortality amongst those with H-AKI alerts (*model 1*), adjusting for age, sex, ethnicity, deprivation, comorbidity score, peak AKI stage, elective or emergency admission and season. Further, we limited model 1 to those with peak AKI stages 2 and 3 in a separate analysis. We then performed a subgroup analysis on patients that had a primary diagnosis of infection (*model 2*).

Standardised mortality ratios (SMRs) were calculated using outputs from our logistic regression model, for hospital trusts in England with more than 10 patients and plotted for the five most common treatment specialty groups in our cohort. Due to the large amount of variation between trusts, the funnel plot is presented with 95% and 99.8% confidence limits.

## Results

**Fig. 1:**
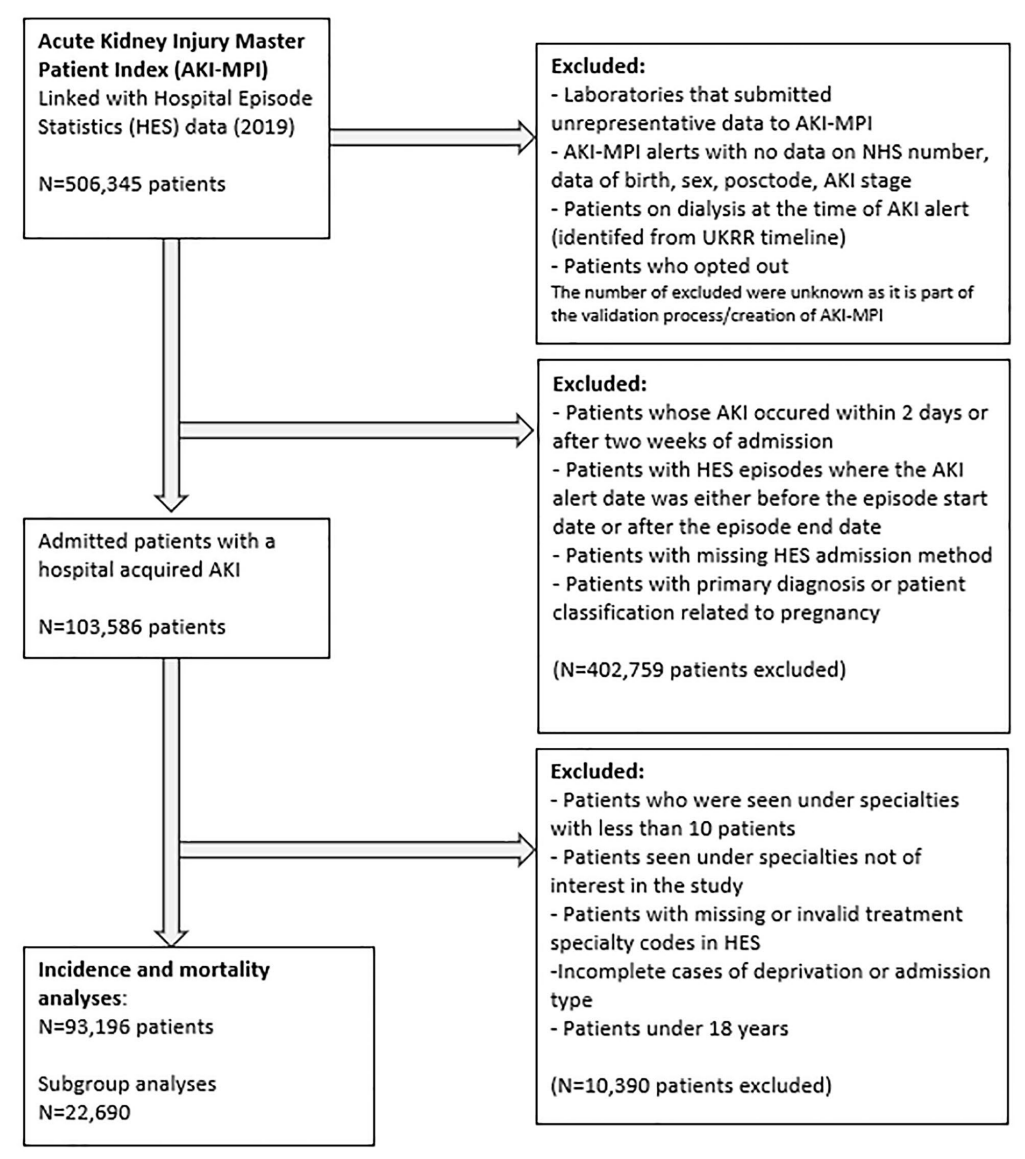
Flow of patient selection

After the application of exclusion criteria, 93,196 patients with H-AKI were eligible for inclusion in the study (Fig. 1). The total number of patients included in the subgroup analyses for patients with H-AKI and ‘infection related primary diagnoses’ was 22,690.

Participant characteristics are displayed in Table 2 for the complete cohort alongside patients with peak AKI stages 2 and 3 only. Regarding the former group, the median age (IQR) was 78.2 years (67.2–86 years), 48.4% were male, 22.4% were in the most deprived quintile and 74.3% had peak AKI stage 1. Demographic and clinical characteristics were similar for the cohort limited to patients with peak AKI stage 2 and 3 only.

**Table 2:** Socio-demographic and clinical characteristics of hospitalized patients with AKI in England in 2019

		All AKI	Peak AKI 2 & 3 only
**Total number of patients** ***N*** **(%)**		93,196 (100)	23,993 (100)
**Age at alert (Median IQR)**		78.2 (67.2–86.0)	76.8 (66.1–85.1)
		***N*** **(%)**	***N*** **(%)**
**Sex**	Male	45,069 (48.4)	12,072 (50.3)
	Female	48,127 (51.6)	11,921 (49.7)
**Ethnicity**	White	77,853 (91.4)	19,950 (91.5)
	Asian	4,042 (4.7)	1,016 (4.7)
	Black	1,772 (2.1)	444 (2)
	Mixed	362 (0.4)	101 (0.5)
	Other	1,178 (1.4)	285 (1.3)
**Deprivation quintile (IMD)**	1 (Least deprived)	16,084 (17.3)	4,019 (16.8)
	2	17,985 (19.3)	4,539 (18.9)
	3	18,735 (20.1)	4,818 (20.1)
	4	19,520 (21)	5,128 (21.4)
	5 (Most deprived)	20,872 (22.4)	5,489 (22.9)
**Reweighted Charlson Comorbidity Index**	CCI = 0	27,390 (29.4)	6,494 (27.1)
	CCI = 1	14,645 (15.7)	3,444 (14.4)
	CCI = 2	19,204 (20.6)	5,008 (20.9)
	CCI > 2	31,957 (34.3)	9,047 (37.7)
**Peak AKI stage**	1	69,203 (74.3)	
	2	14,749 (15.8)	14,749 (61.5)
	3	9,244 (9.9)	9,244 (38.5)
**Hospital setting**	Medical ward	66,189 (71.0)	17,132 (71.4)
	Surgical ward	23,419 (25.1)	5,720 (23.8)
	Acute care setting	1,310 (1.4)	511 (2.1)
	Renal Unit	1,047 (1.1.)	321 (1.3)
	Other	1,231 (1.3)	309 (1.3)
**Admission method**	Emergency	82,835 (88.9)	21,324 (88.9)
	Elective	10,361 (11.1)	2,669 (11.1)
**Season**	Autumn	21,737 (23.3)	5,492 (22.9)
	Spring	24,033 (25.8)	6,099 (25.4)
	Summer	21,531 (23.1)	5,502 (22.9)
	Winter	25,895 (27.8)	6,900 (28.8)
**Primary diagnosis of infection**	No	70,506 (75.7)	17,776 (74.1)
	Yes	22,690 (24.4)	6,217 (25.9)

RCCI - Reweighted Charlson Comorbidity Index score

### Distribution of H-AKI patients in different hospital settings

**Fig. 2:**
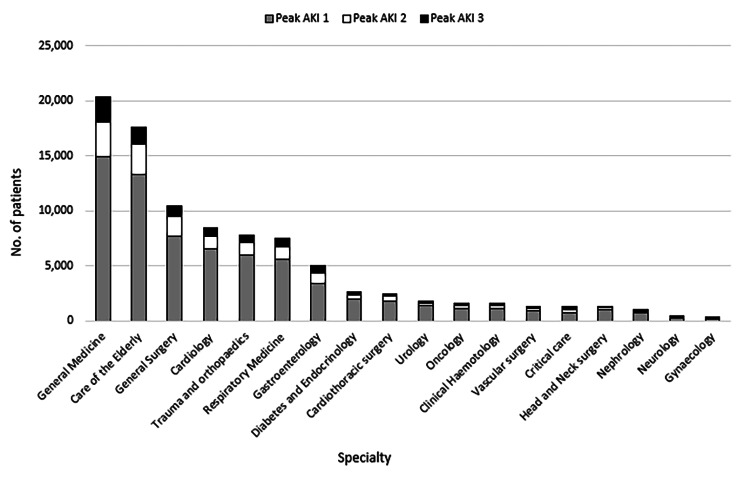
Distribution of AKI episodes across specialties, stratified by peak AKI stage

Distribution of H-AKI patients across each treatment specialty group by peak AKI stage (1,2 and 3) are presented in Fig. 2. Overall, the highest number of H-AKI alerts were detected in patients under the care of medical specialities (71.4%), comprising general medicine (21.9%), care of the elderly (18.9%), cardiology (9.1%), respiratory medicine (8.1%), gastroenterology (5.4%), diabetes and endocrinology (2.9%), oncology (1.8%), haematology (1.7%), nephrology (1.1%), neurology (0.4%) and rheumatology (0.1%). Further, 27.2% of H-AKI developed under the care of surgical specialities; general surgery (11.2%), trauma and orthopaedics (8.4%), cardiothoracic surgery (2.6%), urology (1.9%), vascular surgery (1.4%), head and neck surgery (1.4%) and gynaecology (0.3%). Lastly, 1.4% of all H-AKI cases were identified in patients under critical care. Regarding the severity of AKI, specialities with the highest proportion of peak stage 3 H-AKI (out of all cases of H-AKI observed in that speciality) were nephrology (20.1%), critical care (17.3%) and rheumatology (15.8%).

### H-AKI and 30-day mortality

**Fig. 3:**
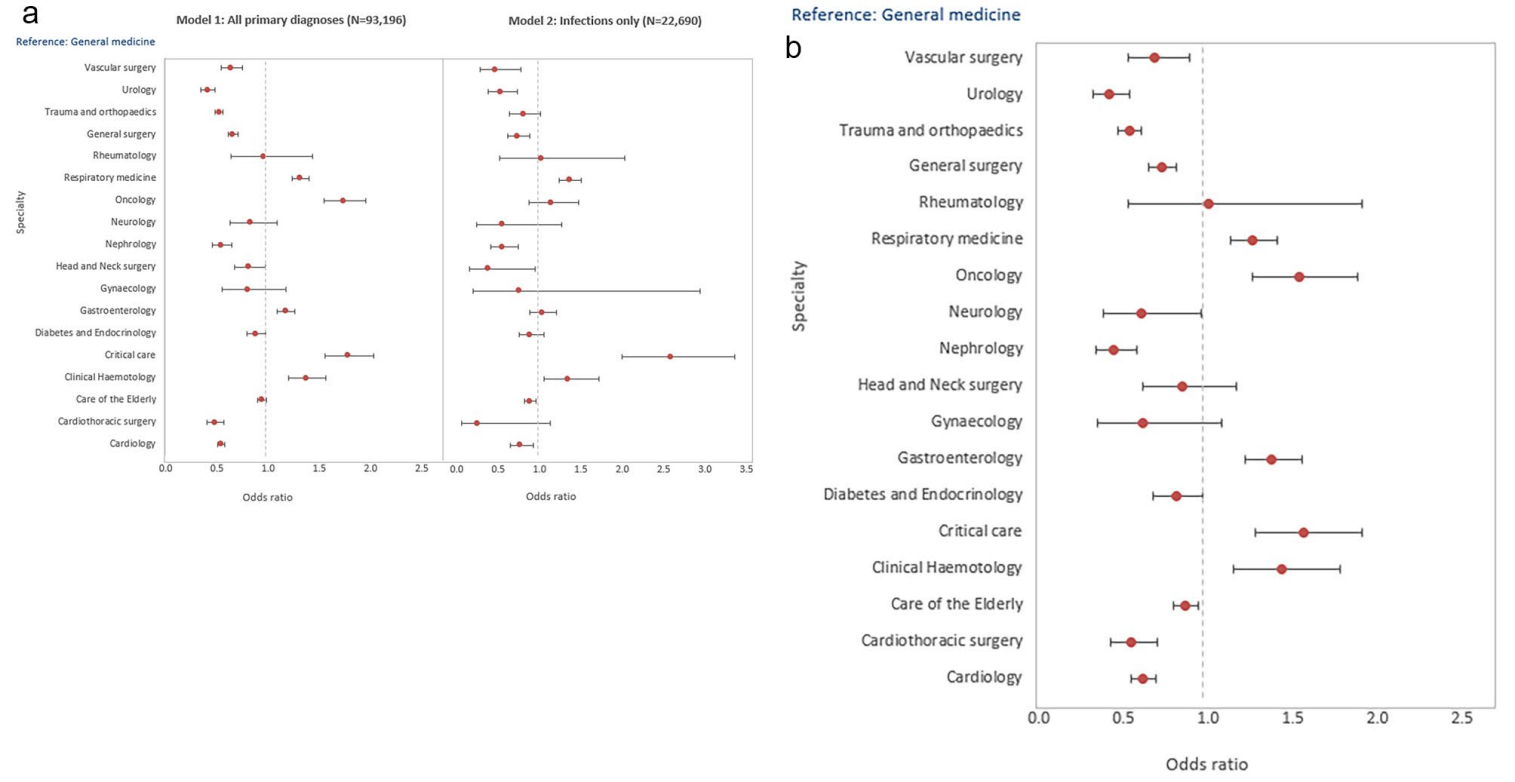
(**a**) Thirty - day mortality by treatment specialty group in patients with a hospital acquired AKI. (**b**) Thirty - day mortality by treatment specialty group in patients with a hospital acquired AKI (peak AKI 2 & 3)

The logistic regression analyses showed a higher 30-day mortality risk for patients with H-AKI who were of male sex, white ethnicity, more deprived, emergency admissions, higher peak AKI stage, higher comorbidity burden and those who acquired their AKI in winter (*Supplementary Table S3*). Despite adjustment for all these factors in our model, mortality risk was significantly lower for patients with H-AKI in many of the surgical specialities when compared to H-AKI patients under general medicine, as shown in Fig. 3a. This included general surgery (OR 0.65, 95% CI 0.61 to 0.7), trauma and orthopaedics (OR 0.52, 95% CI 0.48 to 0.56), urology (OR 0.41, 95% CI 0.35 to 0.48), vascular surgery (OR 0.63, 95% CI 0.54 to 0.75) and cardiothoracic surgery (OR 0.48, CI 0.4 to 0.57). Amongst medical specialities, a lower 30-day mortality risk compared to general medicine was observed for patients who developed H-AKI under the care of cardiology (OR 0.54, 95% CI 0.51 to 0.58) and nephrology (OR 0.54, 95% CI 0.45 to 0.64). 30-day mortality risk compared to general medicine was highest for patients under critical care (OR 1.78, 95% CI 1.56 to 2.03), oncology (OR 1.74, CI 1.54 to 1.96), haematology (OR 1.37, 95% CI 1.2 to 1.57), respiratory medicine (OR 1.31, 95% CI 1.24 to 1.4) and gastroenterology (OR 1.17, 95% CI 1.09 to 1.26). For the analyses restricted to patients with peak AKI 2 and 3 (Fig. 3b), the trends in mortality observed across specialty groups remained similar to those in the complete cohort. Regarding the subgroup analysis for patients with infection related primary diagnoses only, those who were under the care of oncology and gastroenterology no longer had an increased mortality risk when compared to patients seen under general medicine.

### National variation in 30-day mortality for specialities

**Fig. 4:**
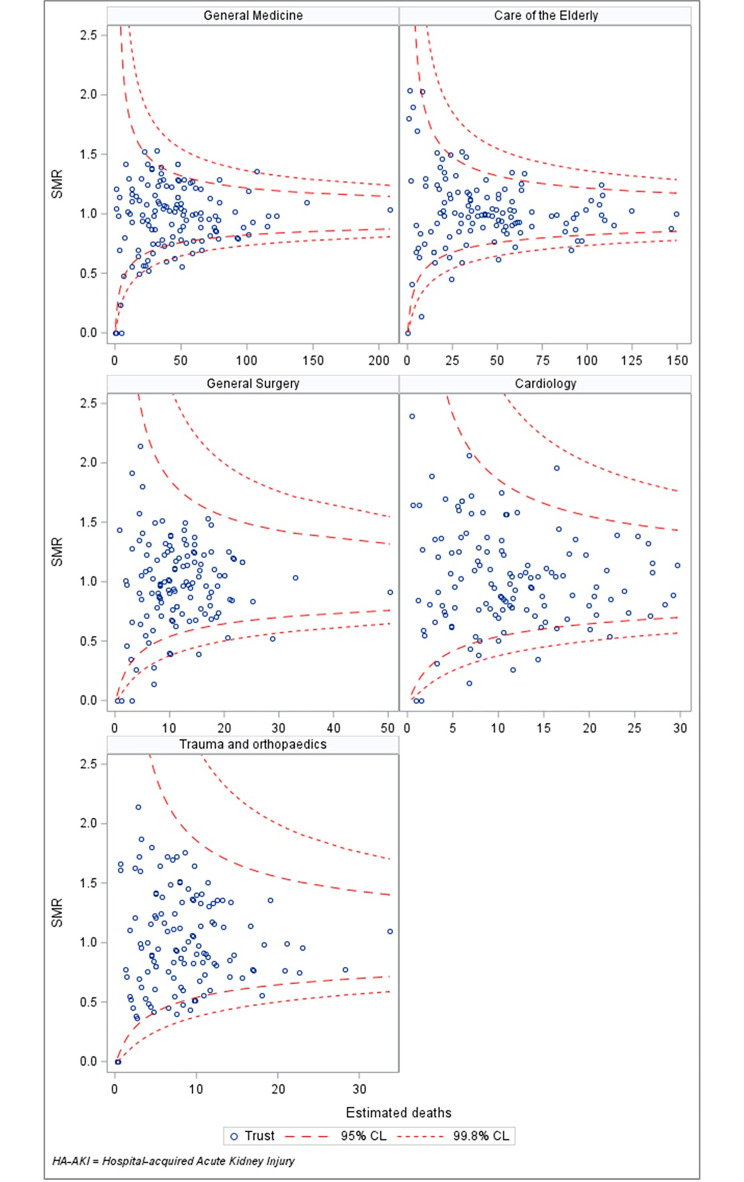
Standardised Mortality Ratios (SMR) by hospital trust for patients with HA-AKI in the 5 largest treatment specialty groups in our cohort Footnote: Funnel plot of SMRs by hospital Trust in the top five largest specialties. Each patient in our cohort was given an individual expected probability of dying, based on the coefficients from the logistic regression model that corresponded to their individual risk factors. The expected probability for each patient was summed across all patients at each Trust to calculate the overall expected number of deaths at that Trust. SMRs were then calculated as the observed number of deaths divided by the expected number of deaths. Owing to the large amount of variation between Trusts, the funnel plot is presented with 95% and 99.8% confidence limits, inflated using an additive random effects model with a 10% trim for overdispersion

Figure 4 shows speciality specific SMRs across different NHS Trusts for the top 5 most common specialty groups in our cohort. For all specialities studied, there were more outlier trusts (95% confidence limits) than would have been expected through chance variation alone. This was 27/127 (general medicine), 18/122 (care of the elderly), 12/127 (general surgery), 13/126 (cardiology) and 11/128 (trauma and orthopaedics).

## Discussion

In this national analysis of over 90,000 patients who developed H-AKI across hospitals in the English NHS, we have demonstrated that H-AKI is a “hospital-wide” concern. The largest burden of H-AKI in our cohort was seen amongst patients under the care of general medicine (21.9%) and care of the elderly (18.9%). Other medical specialities with a significant number of H-AKI cases included cardiology (9.1%), respiratory medicine (8.1%) and gastroenterology (5.4%). Surgical specialities with the largest proportion of H-AKI were general surgery (11.2%) and trauma and orthopaedics (8.4%). Despite accounting for differences in patient case-mix across these specialities, there were significant differences observed in 30-day mortality. The highest mortality risk was noted in patients under critical care, oncology, haematology, gastroenterology and respiratory medicine. Patients with H-AKI under the care of surgical specialities, including trauma and orthopaedics, vascular surgery, urology and cardiothoracic surgery as well as patients under cardiology and nephrology, were noted to have significantly lower 30-day mortality risk compared to patients under general medicine. When comparing speciality specific SMRs for patients with H-AKI across different NHS Trusts, considerable national variation was observed.

### Distribution of H-AKI across specialities

The distribution of H-AKI across specialities described in this study, with the largest burden seen in patients under general medicine, is consistent with previously published analyses from China and Wales and two smaller, single centre studies conducted in English NHS Trusts [5, 26–28].

### 30-day mortality risk across specialities

The specialities with highest 30-day mortality risk following an episode of H-AKI appear to reflect, at least in part, how “unwell” patients are within that speciality e.g. high incidence of patients in critical care with multi-organ failure, high incidence of patients with malignancy and poor prognosis under the care of oncology/ haematology [29–32] Due to limitations of hospital administrative data we were unable to account for differences in severity of acute/ comorbid conditions (recorded just as YES), whether patients were on a palliative care pathway and reverse causality (i.e. patients who were already dying generating an AKI alert). It is also likely that co-morbid medical patients at risk of AKI have diminished physiological reserve to septic or haemodynamic insult [33]. This may not be fully adjusted for by our logistic regression, making direct comparison between medical and surgical patients more challenging. Indeed, such patients admitted while being very unwell with surgical conditions may not be offered surgery and may end up under the care of a physician for supportive care.

The proportion of patients identified as developing H-AKI under the care of nephrology was relatively small, with a lower mortality risk than general medical patients. It is likely that this patient group is highly atypical compared to H-AKI patients across other specialities. Many patients will only be transferred to nephrology for specialist care once they have already developed persistent/severe renal impairment [34, 35]. Our methodology would likely attribute these cases to the speciality that the H-AKI first developed under. For patients who have been identified as developing a H-AKI under nephrology care, it is possible that there is a higher proportion of intrinsic renal disease leading to AKI, compared to other specialities, where there will be more pre-renal AKI and there may also be “selection” of patients for transfer to specialist renal care that are assessed as fit enough for further investigation and management e.g. with kidney biopsy or haemodialysis [34, 36]. These factors may contribute to reduced mortality risk for this group of patients.

To overcome confounding from variations in patients’ primary diagnoses across different specialities, we undertook a subgroup analysis looking at patients with infection related primary diagnoses only. Within this sub-group, many of the mortality trends for H-AKI patients observed for the complete cohort were maintained. Exceptions were patients treated under oncology and gastroenterology, who no longer had an increased mortality risk relative to general medicine. This would suggest that within the overall cohort, highest risk of mortality within these two specialities was for H-AKI patients without infection related primary diagnosis. One challenge in interpreting data related to primary diagnosis group, however, is that patients in hospital settings often have multiple “acute” medical problems and administrative datasets such as HES only permit recording of one primary diagnosis. Future work could focus on further exploration of diagnosis specific mortality rates for patients with AKI, looking at secondary/tertiary diagnosis codes and also procedure codes for surgical admissions.

### National variation in speciality specific 30-day mortality

It is difficult to ascertain from this analysis whether observed variances in patient mortality risk across specialities were due to differences in severity of comorbid conditions that could not be captured in administrative data, physiological differences in AKI “phenotype” or due to variations in patient care. We attempted to explore this further by examining speciality specific 30-day mortality rates for patients with H-AKI across different NHS trusts. Significant national variation was observed for all five of the largest speciality groups i.e., general medicine, care of the elderly, general surgery, cardiology and trauma and orthopaedics. The largest amount of variation was seen for patients under general medicine (27/127 outliers) and care of the elderly (18/122 outliers). Although our model did not adjust for potential differences in complexity of patient’s acute medical conditions i.e., tertiary centres vs. district general hospitals, patients admitted under general medicine and care of the elderly in the UK are likely to present with a similar breadth of clinical problems regardless of the centre that they are presenting to. This unexplained variation warrants further investigation.

### Further strengths and limitations of this study

One of the main strengths of this analysis is the use of a large ‘national’ AKI dataset, where AKI is defined by laboratories “biochemically’, using a standardised national algorithm [14]. We know from previous studies that the MPI captures a much larger population of patients, especially with milder stage 1 AKI than clinical coding in HES alone and can limit some of the bias associated with identifying AKI only through coding of medical records [37].

We limited the analysis to patients with H-AKI as we were interested in comparing AKI care and outcomes across specialities. For patients with community acquired AKI, care is provided mainly in emergency departments and admission units. There would have been multiple confounders in trying to attribute these episodes of AKI and outcomes to a specific speciality.

In this study we have focussed on the overall distribution of H-AKI across specialities for NHS Trusts in England, but we were unable to calculate speciality specific H-AKI incidence rates. This is due to limitations of the available dataset. The UK Renal Registry have access to HES data for patients in the MPI (i.e., those that have triggered a biochemical AKI alert) but we did not have access to data for a comparator population of patients admitted under each speciality that did not trigger an AKI alert. Similarly, we were unable to compare 30-day mortality risk for H-AKI patients in each speciality compared to those who did not develop H-AKI.

It is important to note that the speciality groupings in this analysis have been derived from HES data, based on the speciality of the treating ‘consultant’ (lead clinician) at the time that the AKI episode occurred [38]. In the English NHS, many physicians and surgeons have a generalist component to their work, despite the lead consultant holding sub-speciality accreditation. Often this sub-speciality is used to allocate the ‘treatment speciality’ by clinical coding teams. It is important to note that the structure of the NHS is such that for medical patients in smaller ‘district general hospitals’ especially, a sub-speciality consultant may be delivering care to patients on their ward as a “generalist’ rather than for their speciality specific acute problems. This may confound our findings.

## Conclusion

This is the first national description of patterns in H-AKI distribution and mortality risk across different hospital specialities in the English NHS. The largest burden of H-AKI was found in medical specialities compared to surgical specialities and the highest mortality risk was seen for patients who developed H-AKI in critical care and oncology. Overall, patients who developed H-AKI under surgical specialities had a lower mortality risk than those under general medicine. These described trends can help inform future service planning and quality improvement activity for AKI patients across hospitals in the NHS.

Important limitations of this analysis associated with use of national administrative datasets include possible inconsistencies in coding of treatment specialities and the inability to fully account for differences in severity of patient acute and co-morbid conditions, which are important confounders. Future work may explore diagnosis and procedure specific mortality rates in more detail, rather than across specialities.

## Electronic supplementary material

Below is the link to the electronic supplementary material.

Supplementary Material 1

## Data Availability

The data that support the findings of this study are available from the UK Renal Registry but restrictions apply to the availability of these data, which were used under license for the current study, and so are not publicly available. Data are however available from the authors upon reasonable request and with permission of the UK Renal Registry. Please contact Winnie Magadi at ukrr-research@ukkidney.org if you would like to make a data request.

## Abbreviations

AKI: Acute Kidney Injury
CCI: Charlson Comorbidity Index
CI: Confidence Interval
CKD: Chronic Kidney Disease
CL: Confidence Limit
H-AKI: Hospital-acquired Acute Kidney Injury
HES: Hospital Episodes Statistics
IMD: Index of Multiple Deprivation
KDIGO: Kidney Disease: Improving Global Outcomes
MPI: Master Patient Index
NCEPOD: National Confidential Enquiry into Patient Outcomes and Death
NHS: National Health Service
ONS: Office for National Statistics
RCCI: Re-weighted Charlson Comorbidity Index
RRT: Renal Replacement Therapy
SCr: Serum Creatinine
SHMI: Summary Hospital Mortality Indicator
SMR: Standardised Mortality Ratio
UKRR: UK Renal Registry
