# Supplementary material for: Do outcomes for patients with hospital-acquired Acute Kidney Injury (H-AKI) vary across specialties in England?
Source: BMC Nephrol. 2023 Jun 29;24:193. doi: 10.1186/s12882-023-03197-z (PMC10308766; doi:10.1186/s12882-023-03197-z)
Supplement: Supplementary file 1 — Supplementary Material 1 [file 12882_2023_3197_MOESM1_ESM.pdf]

Supplementary Materials

Supplementary Table S1: Re-weighted Charlson comorbidity Index (RCCI) using all hospitalised AKI patients in England

COMORBIDITY	AKI REWEIGHTED CCI
MI	0
CEREBROVASCULAR DISEASE	1
HEART FAILURE	2
RHEUMATIC DISEASE	0
DEMENTIA	1
DIABETES	0
MILD LIVER DISEASE	2
PEPTICULCER DISEASE	1
PERIPHERAL VASCULAR DISEASE	1
CHRONIC PULMONARY DISEASE	1
MALIGNANCY	2
DIABETES COMPLICATIONS	0
PARAPLEGIA	1
RENAL DISEASE	0
MALIGNANCY METASTATIC SOLID TUMOUR	3
MEDIUM OR SEVERE LIVER DISEASE	3

*Adjusted for age, sex, level of AKI alert, month of AKI alert, and each comorbidity group individually

Supplementary Table S2: Type of infection by treatment specialty group in AKI patients with infections as a primary diagnosis

	**Summary Hospital-level Mortality Indicator (SHMI) diagnosis groups (%)**														
**Specialty group**	**Group 1**	**Group 2**	**Group 3**	**Group 4**	**Group 5**	**Group 6**	**Group 7**	**Group 8**	**Group 9**	**Group 10**	**Group 11**	**Group 12**	**Group 13**	**Group 14**	**Total (N)**
**General Medicine**	0.1	22.5	0.6	0.9	1.4	40.6	6.6	4.6	0.7	0.3	12.9	7.7	1.0	0.1	**7,337**
**Care of the Elderly**	0.0	18.9	0.4	0.9	1.0	40.5	7.3	3.7	0.0	0.1	19.6	6.8	0.8	0.1	**6,135**
**Respiratory Medicine**	0.2	12.2	0.2	0.4	0.7	71.0	6.8	1.4	0.1	0.1	4.2	2.6	0.4	0.0	**3,147**
**Gastroenterology**	0.0	31.0	0.7	2.4	0.9	19.7	3.5	12.6	12.0	2.4	8.4	6.3	0.1	0.2	**1,056**
**General Surgery**	0.2	26.3	0.4	1.2	0.1	6.5	0.8	8.6	15.6	24.9	4.3	9.3	1.3	0.8	**1,036**
**Diabetes and Endocrinology**	0.0	24.3	0.8	0.9	1.8	30.8	5.9	5.7	0.0	0.1	14.9	11.1	3.6	0.1	**855**
**Cardiology**	0.0	22.6	1.6	0.8	0.3	50.9	8.2	2.0	0.4	0.1	7.7	4.9	0.5	0.0	**754**
**Trauma and orthopaedics**	0.4	17.3	0.4	0.0	1.7	14.8	0.6	0.9	0.0	0.0	2.5	23.9	37.0	0.4	**473**
**Clinical Haematology**	0.0	60.1	0.8	2.5	0.0	23.5	4.9	3.3	0.6	0.0	1.1	2.2	0.0	1.1	**366**
**Oncology**	0.0	48.1	1.9	1.6	0.3	25.6	6.7	5.8	0.0	1.6	4.2	3.9	0.0	0.3	**312**
**Nephrology**	0.0	24.1	1.0	2.0	0.0	33.6	6.5	5.2	0.3	0.3	17.6	8.8	0.7	0.0	**307**
**Critical care**	1.3	26.6	0.7	1.3	2.0	53.8	2.3	2.7	3.3	3.0	2.0	0.7	0.3	0.0	**301**
**Urology**	0.0	38.7	0.7	0.0	0.4	4.5	0.0	0.4	0.0	0.4	53.5	0.7	0.4	0.4	**269**
**Vascular surgery**	0.0	21.8	1.4	0.0	0.0	7.0	0.0	0.7	0.0	0.0	0.0	22.5	40.1	6.3	**142**
**Head and Neck surgery**	5.4	19.6	0.0	1.8	25.0	19.6	1.8	0.0	0.0	0.0	1.8	10.7	8.9	5.4	**56**
**Neurology**	2.0	9.8	0.0	7.8	58.8	5.9	2.0	0.0	0.0	0.0	11.8	0.0	2.0	0.0	**51**
**Rheumatology**	0.0	17.4	0.0	4.4	2.2	28.3	6.5	6.5	0.0	0.0	23.9	6.5	4.4	0.0	**46**
**Gynaecology**	0.0	45.8	4.2	0.0	0.0	8.3	0.0	0.0	0.0	16.7	25.0	0.0	0.0	0.0	**24**
**Cardiothoracic surgery**	0.0	26.1	4.4	0.0	0.0	56.5	4.4	0.0	0.0	0.0	0.0	0.0	8.7	0.0	**23**

SHMI Diagnosis Groups Labels
Group 1: Tuberculosis
Group 2: Septicaemia (except in labour), Shock
Group 3: Bacterial infection; unspecified site
Group 4: Non-HIV related infections
Group 5: Central nervous system infections
Group 6: Pneumonia (excluding TB/STD)
Group 7: Acute bronchitis
Group 8: Intestinal infection
Group 9: Regional enteritis and ulcerative colitis
Group 10: Appendiceal conditions, Peritonitis & intestinal abscess
Group 11: Urinary tract infections
Group 12: Skin and subcutaneous tissue infections
Group 13: Infective arthritis and osteomyelitis (excluding TB/STD)
Group 14: Lymphadenitis, Gangrene

Supplementary Table S3: Risk factors for 30-day mortality in patients with a hospital acquired AKI in England

Variable	MODEL 1 - All SHMI primary diagnoses<br>OR (95% CI)	MODEL 2 - Infections<br>OR (95% CI)
**Treatment specialty (ref: General Medicine)**		
Cardiology	0.54 (0.51-0.58)	0.77 (0.65-0.92)
Cardiothoracic Surgery	0.48 (0.4-0.57)	0.25 (0.06-1.13)
Care of the Elderly	0.94 (0.89-0.99)	0.89 (0.82-0.96)
Clinical Haematology	1.37 (1.2-1.57)	1.35 (1.05-1.72)
Critical care	1.78 (1.56-2.03)	2.59 (1.99-3.36)
Diabetes and Endocrinology	0.88 (0.8-0.97)	0.89 (0.75-1.05)
Gastroenterology	1.17 (1.09-1.26)	1.03 (0.88-1.21)
Gynaecology	0.8 (0.55-1.17)	0.76 (0.2-2.94)
Head and Neck surgery	0.81 (0.67-0.98)	0.38 (0.15-0.94)
Nephrology	0.54 (0.45-0.64)	0.55 (0.41-0.74)
Neurology	0.83 (0.63-1.09)	0.55 (0.24-1.26)
Oncology	1.74 (1.54-1.96)	1.14 (0.88-1.47)
Respiratory Medicine	1.31 (1.24-1.4)	1.37 (1.24-1.5)
Rheumatology	0.95 (0.63-1.44)	1.02 (0.51-2.03)
General Surgery	0.65 (0.61-0.7)	0.74 (0.61-0.88)
Trauma and Orthopaedics	0.52 (0.48-0.56)	0.8 (0.64-1.01)
Urology	0.41 (0.35-0.48)	0.53 (0.38-0.74)
Vascular Surgery	0.63 (0.54-0.75)	0.47 (0.28-0.78)
**Age at alert**	1.04 (1.04-1.04)	1.04 (1.04-1.05)
**Gender (ref: Male)**		
Female	0.76 (0.73-0.78)	0.72 (0.68-0.76)
**Ethnicity (ref:(White)**		
Asian	0.81 (0.74-0.88)	0.7 (0.58-0.83)
Black	0.7 (0.61-0.8)	0.86 (0.67-1.12)
Mixed	0.79 (0.58-1.06)	0.68 (0.38-1.24)

Other	0.82 (0.7-0.96)	0.8 (0.58-1.1)
Missing	1.12 (1.06-1.19)	1.01 (0.9-1.13)
**Deprivation quintile (ref: 1-Least deprived)**		
Quintile 2	1.03 (0.97-1.08)	1.07 (0.97-1.18)
Quintile 3	1.02 (0.97-1.08)	1.07 (0.97-1.19)
Quintile 4	1.06 (1-1.12)	1.12 (1.02-1.24)
Quintile 5	1.09 (1.04-1.15)	1.21 (1.1-1.33)
**Peak AKI stage (ref: Stage 1)**		
Stage 2	2.56 (2.46-2.67)	2.47 (2.28-2.67)
Stage 3	3.87 (3.69-4.07)	3.85 (3.5-4.22)
**Modified charlson comorbidity index**	1.27 (1.26-1.28)	1.25 (1.23-1.27)
**Type of admission method (ref: Emergency)**		
Elective	0.36 (0.33-0.39)	0.74 (0.57-0.96)
**Season (ref: Summer)**		
Autumn	1.05 (1-1.1)	1.05 (0.96-1.16)
Spring	1.06 (1.01-1.11)	1.07 (0.98-1.17)
Winter	1.2 (1.14-1.26)	1.21 (1.11-1.32)
**Infection as primary diagnosis (ref:No)**		
Yes	1.33 (1.28-1.38)	

SHMI- Summary Hospital-level Mortality Indicator
